# Metabolomic Profiling Identified Serum Metabolite Biomarkers and Related Metabolic Pathways of Colorectal Cancer

**DOI:** 10.1155/2021/6858809

**Published:** 2021-12-07

**Authors:** Chengjian Zhang, Shengnan Zhou, Huijing Chang, Feng Zhuang, Yang Shi, Le Chang, Wanchao Ai, Juan Du, Wei Liu, Humin Liu, Xukun Zhou, Zhong Wang, Tao Hong

**Affiliations:** ^1^General Surgery Department, Hospital of Xinjiang Production and Construction Corps, Urumchi, China; ^2^General Surgery Department, Peking Union Medical College Hospital, China Academy of Medical Science & Peking Union Medical College, Beijing, China; ^3^Department of Gastrointestinal Surgery, National Cancer Center/National Clinical Research Center for Cancer/Cancer Hospital & Shenzhen Hospital, Chinese Academy of Medical Sciences and Peking Union Medical College, Shenzhen, China

## Abstract

**Background:**

The screening and early detection of colorectal cancer (CRC) still remain a challenge due to the lack of reliable and effective serum biomarkers. Thus, this study is aimed at identifying serum biomarkers of CRC that could be used to distinguish CRC from healthy controls.

**Methods:**

A prospective 1 : 2 individual matching case-control study was performed which included 50 healthy control subjects and 98 CRC patients. Untargeted metabolomic profiling was conducted with liquid chromatography tandem mass spectrometry (LC-MS/MS) to identify CRC-related metabolites and metabolic pathways.

**Results:**

In total, 178 metabolites were detected, and an orthogonal partial least-squares-discriminant analysis (OPLS-DA) model was useful to distinguish CRC patients from healthy controls. Nine metabolites showed significantly differential serum levels in CRC patients under the conditions of variable importance in projection (VIP) > 1, *p* < 0.05 using Student's *t*-test, and fold change (FC) ≥ 1.2 or ≤0.5. The above nine metabolites were 3-hydroxybutyric acid, hexadecanedioic acid, succinic acid semialdehyde, 4-dodecylbenzenesulfonic acid, prostaglandin B2, 2-pyrocatechuic acid, xanthoxylin, 12-hydroxydodecanoic acid, and formylanthranilic acid. Four potential biomarkers were identified to diagnose CRC through ROC curves: hexadecanedioic acid, 4-dodecylbenzenesulfonic acid, 2-pyrocatechuic acid, and formylanthranilic acid. All AUC values of these four serum biomarkers were above 0.70. In addition, the exploratory analysis of metabolic pathways revealed the activated states for the vitamin B metabolic pathway and the alanine, aspartate, and glutamate metabolic pathways associated with CRC.

**Conclusion:**

The 4 identified potential metabolic biomarkers could discriminate CRC patients from healthy controls, and the 2 metabolic pathways may be activated in the CRC tissues.

## 1. Introduction

Colorectal cancer (CRC) is one of the most common malignant tumours of the digestive system, and the morbidity (10.0%) and mortality (9.4%) of it rank third and second around the world, respectively [1]. Because of the late diagnosis and advanced disease, 30%-50% of CRC patients still cannot survive for more than 5 years among high-income countries [2].

The screening and early diagnosis of CRC have been shown to improve the prognosis [3]. However, the current screening tests such as serum tumour markers and faecal occult blood tests have low specificity and low sensitivity and are not valid as the early diagnostic screening. Colonoscopy or sigmoidoscopy is not easy to popularize as a screening method among the large-scale population, because it is an invasive examination with relatively high expense, poor compliance, and a certain risk of complications, including postoperative bleeding (0.26%), perforation (0.05%), and death (2.9/100,000) [4]. Therefore, the discovery of novel meaningful biomarkers is important in the early diagnosis of CRC.

Currently, circulating biomarkers, including circulating tumour DNA (ctDNA) [5], microRNAs (miRNAs) [6], and metabolites [7, 8], are a research focus to diagnose CRC early and improve prognosis. Dysregulated metabolism is one of the hallmarks of cancer, and the overexpression or loss of genes can cause changes in metabolism [9, 10]. In addition, the metabolic profiles of cancer cell changes are associated with disease progression [11]. Thus, the assessment of endogenous small-molecule metabolism is forming as promising minimally invasive biomarkers for the early diagnosis of cancers.

Metabonomics [12] is a new branch of systems biology that has emerged after genomics, transcriptomics, and proteomics and can measure the levels of many small-molecule metabolites in biospecimens. Major technologies that are used for metabolomics include nuclear magnetic resonance (NMR) [13] and mass spectrometry (MS) [14]. Previous studies used the NMR-based metabolomics to screen out some circulating metabolites to distinguish CRC patients from those without cancer [15, 16]; however, NMR has the weaknesses of low sensitivity and a narrow detection dynamic range. Therefore, the current research found that the circulating xanthine, hypoxanthine, and D-mannose levels could be the biomarkers for CRC patients by using more sensitive technology [17], such as gas chromatography-MS (GC-MS) and liquid chromatography tandem MS (LC-MS/MS). However, most of these studies ignored factors that could affect the metabolism, such as age, sex, body mass index (BMI), and diet [18].

This study is aimed at identifying circulating biomarkers of CRC disease through the metabolomic profiling of human serum samples by using LC-MS/MS, which may offer new methods for the early diagnosis and screening of CRC disease.

## 2. Materials and Methods

### 2.1. Participants

This was a prospective 1 : 2 individual matching case-control study that included two groups: the healthy control group and the CRC group recruited at the Peking Union Medical College Hospital between 2019 and 2021. The inclusion criteria of the healthy control group were healthy adults over 18 years of age with no forms of cancer. The inclusion criteria of the CRC group were as follows: over 18 years old, a definite diagnosis of carcinoma by biopsy pathology through colonoscopy or proctoscopy, and no metabolic diseases such as diabetes, fatty liver, or obesity. The research was performed in compliance with the 1964 Helsinki Declaration and its later amendments of ethical standards. Moreover, the participants of the CRC group were selected based on matched age, sex, and BMI in the control group, thus decreasing these effects. The study protocol was ethically reviewed and approved by the Institutional Review Board of Peking Union Medical College Hospital. All participants involved in this study have signed informed consent.

### 2.2. Study Samples

Demographic information, including age, gender and BMI, and medical history, of all enrolled participants was recorded for subsequent analysis. All blood samples of the participants were collected in the morning after fasting for 8-12 hours, and separated serum was used for the following analysis on the day of sample collection within 2 h and preserved at -80°C.

### 2.3. Sample Testing and Data Preprocessing

All samples were subjected to metabolic analysis by an LC-MS/MS platform (Q Exactive Orbitrap, Thermo Fisher Scientific, USA). Briefly, the serum samples were thawed at 4°C for approximately 2 hours, 150 *μ*l serum was taken, 450 *μ*l precooled formaldehyde was added, and the samples were placed at -20°C for 1 hour after vortexing. Then, the mixed solution was centrifuged at 14000 rpm for 15 minutes at 4°C. The resulting supernatants were transferred to LC-MS/MS for analysis [19].

LC-MS/MS analyses were performed using an UHPLC system (Vanquish, Thermo Fisher Scientific) with a UPLC BEH Amide column (2.1 mm × 100 mm, 1.7 *μ*m) coupled to the Q Exactive HFX mass spectrometer (Orbitrap MS, Thermo). The mobile phase consisted of 25 mmol/L ammonium acetate and 25 ammonia hydroxide in water (pH = 9.75) (A) and acetonitrile (B). The autosampler temperature was 4°C, and the injection volume was 3 *μ*L.

The QE HFX mass spectrometer was used for its ability to acquire MS/MS spectra on the information-dependent acquisition (IDA) mode in the control of the acquisition software (Xcalibur, Thermo). In this mode, the acquisition software continuously evaluates the full scan MS spectrum. The ESI source conditions were set as the following: sheath gas flow rate as 30 Arb, Aux gas flow rate as 25 Arb, capillary temperature 350°C, full MS resolution as 60000, MS/MS resolution as 7500, collision energy as 10/30/60 in NCE mode, and spray voltage as 3.6 kV (positive) or -3.2 kV (negative), respectively. The MetaboScape 3.0 software (Bruker, USA) was used to preprocess the data of the sample quality spectra, such as peak extraction, denoising, and normalization.

### 2.4. Statistical Analysis

The identified data were analysed by SIMCA software (V16.0.2, Sartorius Stedim Data Analytics AB, Umea, Sweden). Principal component analysis (PCA) is an unsupervised mode that can reduce the dimensionality of data and distinguish the internal characteristics of data by several main components. PCA was first performed to estimate the discreteness of the data and the classification of the samples. Then, orthogonal projections to latent structures-discriminant analysis (OPLS-DA) was used to understand the metabolic changes between healthy controls and CRC patients. The permutation test with 200 iterations was implemented to assess the statistical significance and prevent overfitting of the OPLS-DA model. It is generally believed that the intercept of *R*^2^ on the *Y*-axis is less than 0.4 and the intercept of *Q*^2^ on the *Y*-axis is less than 0, which indicates that the model does not exhibit overfitting and has good robustness.

To avoid false positive errors caused by the multivariate statistics, we also performed the univariate analyses: Student's *t*-test and fold change (FC) analysis. Therefore, the candidate metabolites were selected based on the variable importance in the projection (VIP) values, which were considered responsible for group discrimination of OPLS-DA more than 1, the *p* value of Student's *t*-test less than 0.05, and the cut-off value of FC 0.5 or 1.2. ROC curves were performed to estimate the ability of metabolites to distinguish the CRC patients from healthy people. The true positive rate can be represented by sensitivity as the vertical coordinate, and the false positive rate can be represented by 1-specificity as the horizontal coordinate. The area under the ROC curve (AUC) can be used to determine the accuracy of the metabolite in distinguishing the two different groups. AUC = 0.5 indicates that the metabolite is not useful in distinguishing; 0.7 ≤ AUC ≤ 0.9 indicates that the metabolite has relatively high accuracy; AUC > 0.9 indicates high accuracy.

In addition, the selected metabolites were mapped into the KEGG database for a comprehensive analysis, including pathway enrichment analysis and topological analysis, which can be presented by the bubble plot. Each bubble in the bubble plot represents a metabolic pathway, and the *X*-axis and bubble size represent the impact factor of topological analysis, and the *Y*-axis and bubble colour indicate the *p* value of the enrichment analysis.

## 3. Results

### 3.1. Study Population Characteristics

Our study included 50 healthy participants and 98 CRC patients.  Table 1 presents the demographic characteristics of the 2 groups, including the gender, age, BMI value, and level of serum carcinoma embryonic antigen (CEA). There were no significant differences between the CRC group and the control healthy group in the variables of age (61.61 vs. 61.52, *p* = 0.955) and BMI (24.56 vs. 25.31, *p* = 0.309). Among the 98 CRC patients, 41 patients were diagnosed with colon cancer, and 57 patients were diagnosed with rectal cancer. In addition, the mean value of CEA of CRC patients was 8.91 mmol/L which is larger than that of the control group, but not statistically significant.

### 3.2. Metabolites Expressed in the CRC Group and Control Group

**Figure 1:**
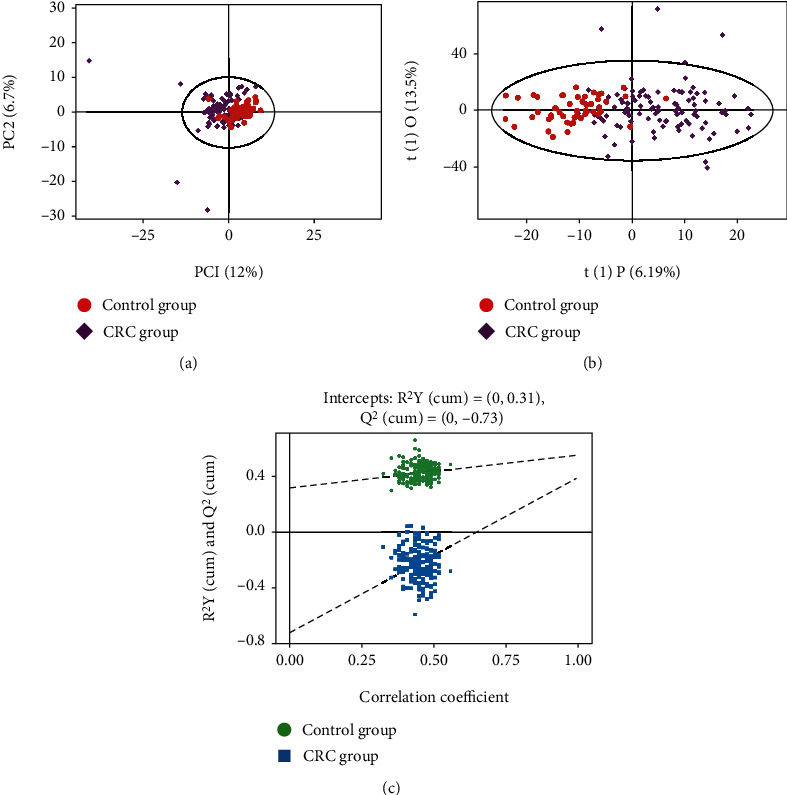
(a) The PCA performed on two groups: 98 CRC patients (purple dots) and 50 healthy controls (red dots). (b) The OPLS-DA model was constructed using data from 98 CRC patients (purple dots) and 50 healthy controls (red dots). (c) The permutation test plot of OPLS-DA (permutation test with 200 times, *p* value CV-ANOVA = 0.004); the green dots represent the value of *R*^2^*Y*, and the blue dots represent the value of *Q*^2^.

In total, 178 metabolites were detected by LC-MS. The PCA performed on all samples revealed that the healthy control samples were tightly clustered in the PCA score plots (Figure 1(a)); however, the CRC samples were fairly dispersed. Moreover, the metabolic profiles of the CRC group and control group were not well distinguished. Further OPLS-DA score plots (Figure 1(b)) show a clear separation between the CRC group and the control group. The permutation test assures the validity of the OPLS-DA model with all permuted *Q*^2^ and *R*^2^ values lower than the original values, and the *Q*^2^ (cum) intercepted the *Y*-axis at -0.73 (Figure 1(c)).

### 3.3. Discovery and Identification of Different Metabolites

Through the multivariate statistical analysis, we successfully established OPLS-DA models for intergroup differentiation and identified significant metabolic differences between the CRC group and the control group. The significantly altered metabolites were selected as biomarker candidates with Student's *t*-test (*p* < 0.05) and the VIP threshold (VIP > 1) in the aforementioned OPLS-DA model; FC > 1.2 or <0.5 was also the screening index. In total, 9 metabolites were identified as the potential biomarkers and are summarized in Table 2. Among the levels of these differential metabolites in CRC patients compared to the control group, 2 metabolites were downregulated, and 7 metabolites were upregulated, including hydroxy acids, fatty acyls, benzene, and substituted derivatives and organooxygen compounds.

### 3.4. Metabolic Biomarkers of Diagnostic Value

**Figure 2:**
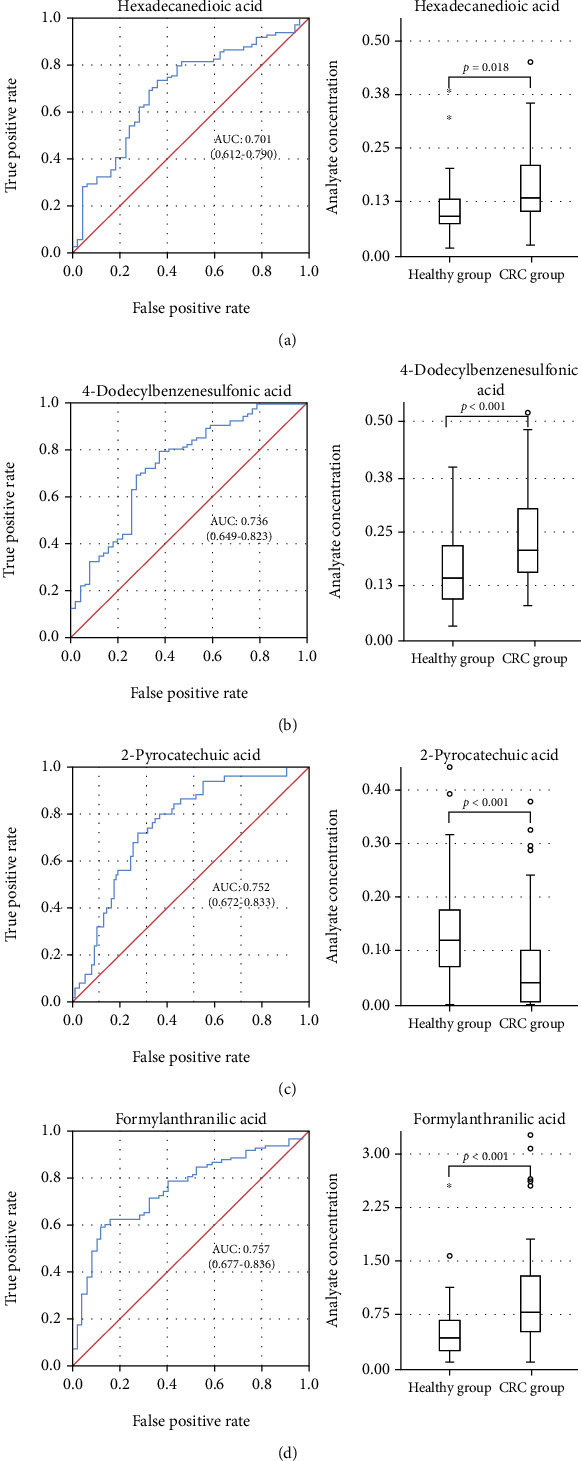
ROC curves and box plots of the identified 4 potential biomarkers: (a) hexadecanedioic acid; (b) 4-dodecylbenzenesulfonic acid; (c) 2-pyrocatechuic acid; (d) formylanthranilic acid.

The diagnostic potential of these 9 identified metabolites for CRC patients was evaluated by the ROC curve analysis. The AUC was used to test the reliability of the differential metabolites. The metabolites with AUC values > 0.7 were hexadecanedioic acid, 4-dodecylbenzenesulfonic acid, 2-pyrocatechuic acid (2,3-dihydroxybenzoic acid (2,3-DHBA)), and formylanthranilic acid, as shown in Figure 2). These four metabolites have high diagnostic value in the diagnosis of colorectal cancer. Compared with the healthy group, hexadecanedioic acid, 4-dodecylbenzenesulfonic acid, and formylanthranilic acid levels increased in CRC patients. In contrast, the 2-pyrocatechuic acid level decreased in CRC patients (Figure 2). It was proven that these four metabolites had a high diagnostic value in the diagnosis of CRC.

### 3.5. Metabolic Pathway Analysis of Differential Metabolites

**Figure 3:**
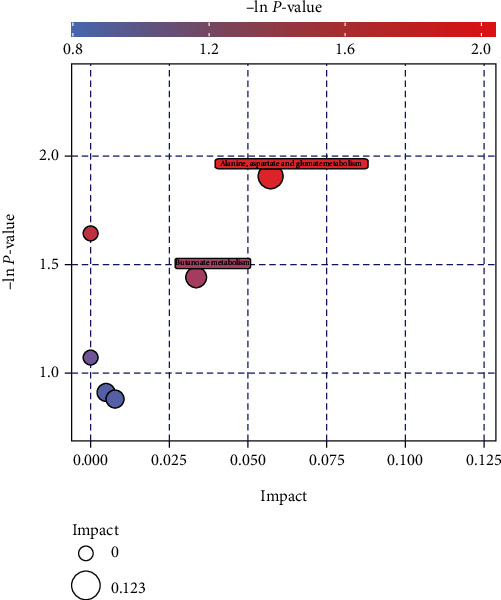
Bubble blot of pathway analysis.

Through enrichment analysis and topological analysis of the pathway where the selected differential metabolites are located, we can further screen the metabolic pathway and find the key pathway with the highest correlation with the differential metabolites. According to the KEGG database, 2 metabolic pathways can be matched, which suggests that these pathways may be involved in the metabolic network of colorectal carcinoma, as shown in Figure 3. This metabolic pathway information will provide directions for subsequent research.  Table 3 shows the results of the topological analysis and enrichment analysis of differential metabolites.

## 4. Discussion

In this study, we aimed to discover and identify serum metabolite biomarkers for the early diagnosis of colorectal cancer based on the metabonomics analysis of CRC patients in comparison with healthy people using the LC-MS platform. In addition, the related metabolic pathways were recognized.

Our results showed that 9 metabolites were significantly altered in CRC patients compared to healthy people; among these differential metabolites, hexadecanedioic acid, 4-dodecylbenzenesulfonic acid, 2-pyrocatechuic acid, and formylanthranilic acid have certain diagnostic values. To our knowledge, our study is the first to display the different serum metabolomics between CRC patients and healthy people after adjusting for gender, age, and BMI, which can influence the metabolism. Notably, we observed that 2 metabolic pathways were associated with CRC patients, alanine, aspartate, and glutamate metabolism and vitamin B6 metabolism.

Hexadecanedioic acid, which is a type of long-chain dicarboxylic acid, was activated by the mitochondrial and microsomal fractions [20]. Our results indicated that the rise of plasma hexadecanedioic acid could be utilized as a potential signature for CRC diagnosis, but the causal relationship between them is unclear. To our knowledge, hexadecanedioic acid has been demonstrated to exhibit antimycotic activity [21, 22] in few studies. Some metabolites from the fungus can inhibit cancer cell growth and migration [23] and induce cancer cell apoptotic death [24] in vitro. A previous study [25] also reported that hexadecanedioic acid was one of the identified biomarkers of the diagnostic panel for the early-stage CRC patients; however, it showed a decreasing trend compared to healthy individuals. The downregulation of hexadecanedioic acid may be caused by the larger energy requirements for cancer cell proliferation because it is consumed by the mitochondria through beta oxidation to produce ATP to supply energy for the body. Whether hexadecanedioic acid is a condition for the occurrence of colorectal cancer or a phenomenon after tumours have occurred requires further study and discussion.

The differential metabolite with the highest diagnostic accuracy for colorectal cancer in our study was formylanthranilic acid, which was upregulated in CRC patients compared with healthy controls. Formylanthranilic acid is the metabolite of tryptophan by the kynurenine pathway [26]. The increased level of formylanthranilic acid suggests the activity of the tryptophan-kynurenine metabolic pathway. Studies have shown that the tryptophan-kynurenine pathway plays a crucial role in promoting colorectal cancer progression [27, 28]. In addition, colon cancer cells display greater uptake and processing of tryptophan than normal colonic cells and tissues [29]. Formylanthranilic acid was first reported as a serum biomarker with diagnostic potential in CRC patients by our study. Its elevation may be due to the increased uptake and consumption of tryptophan by tumour cells, combined with the activation of tryptophan-kynurenine metabolic pathways. Meanwhile, the upregulation of formylanthranilic acid proves that the tryptophan metabolism plays an important role in the development of colon cancer [30], and tryptophan metabolism along the kynurenine pathway can be a particularly promising target for future immunotherapy [31]. Another biomarker of upregulated serum levels is 4-dodecylbenzenesulfonic acid, which is a derivative of benzenesulfonic acids, and the main metabolic pathways in which it participates are unclear. A recent study reported benzenesulfonic acid derivatives as human neutrophil elastase inhibitors to treat acute respiratory distress syndrome [32]; however, there is still no research related to the tumour metabolic pathways involving benzenesulfonic acids. This topic can be a future direction of research on the pathogenesis of colorectal cancer.

Our study found that the only diagnostic metabolic biomarker with a downregulated trend in CRC patients compared to healthy controls was 2,3-DHBA. As a metabolite of aspirin and salicylic acid, it has been shown to inhibit colon cancer cell growth [33, 34]. A consensus has not been reached on the anticancer mechanisms of 2,3-DHBA. Some studies have demonstrated that the aspirin metabolite 2,3-DHBA can inhibit cyclin-dependent kinase enzyme activity and cancer cell growth [35]. Another mechanism may be due to its antioxidant properties, since it is an effective scavenger of free radicals and reactive nitrogen species [36, 37]. Even without taking aspirin or salicylic acid, 2,3-DHBA compounds are present as normal constituents of serum [38], which may arise from diet. Interestingly, salicylic acid has been widely found in many foods, mainly fruits and vegetables [39]. The decrease in 2,3-DHBA in CRC patients in our study may be caused by the low intake of vegetables and fruits, which can support the current hot viewpoint that a high-fat and low-fibre diet may increase the risk of colon cancer incidence [40, 41].

Significant changes of other serum metabolites were also observed in CRC patients, including upregulated levels of 3-hydroxybutyric acid, succinic acid semialdehyde, prostaglandin B2, and 12-hydroxydodecanoic acid and downregulated levels of xanthoxylin. All of these metabolites for CRC obtained from the untargeted metabolic analysis are reported in our study for the first time and guide our focus on two main metabolic pathways that may play critical roles in colorectal cancer: the vitamin B6 metabolic pathway and the alanine, aspartate, and glutamate metabolism pathways. Both pathways contain succinic acid semialdehyde. Vitamin B6 has been found to be related to colorectal cancer risk [42]. Pyridoxine is the metabolite of the vitamin B6 metabolic pathway, and there was a negative correlation between serum pyridoxine level and the risk of CRC [43, 44]. Pyridoxine is converted to succinic semialdehyde in the vitamin B metabolic pathway [45]. The increased succinic acid semialdehyde level suggests that the vitamin B metabolic pathway is an activated state in colorectal cancer tissue, which decreases pyridoxine with protective effects. Moreover, the bubble diagram suggested that pathways involving metabolism of alanine, aspartate, and glutamate changed dramatically in CRC cases, which has been selected as one of the disturbed metabolic pathways in mice with colon cancer in an orthotopic transplantation model (46). In human colorectal cancer cells, alanine, aspartic acid, and proline were converted to glutamic acid by the stable isotope technique [46], and an increased level of serum glutamate was also detected in colorectal cancer cases [47]. Then, glutamate is converted to *γ*-aminobutyric acid (GABA) by the rate-limiting enzyme glutamate decarboxylase 1 (GAD1), which is overexpressed in tumour tissues [48, 49]. In the final step of the GABA shunt pathway, succinic semialdehyde is oxidized to succinate [50]. Thus, we suspect that the alanine, aspartate, and glutamate metabolic pathways were also activated, and most metabolites involved in this metabolic pathway were elevated in colorectal cancer, and the important key enzymes in the pathway may be the therapeutic targets.

There are some limitations to our study. First, all the included CRC patients were diagnosed with biopsy results under endoscopy, and the exact tumour stages that could influence the serum metabolites were deficient. Later research can be based on a larger sample size, grouping tumour stages and comparing the differences in serum metabolites among the groups. Second, these identified metabolites with potential diagnostic value must be validated in large-scale external cohorts with multicentre investigations. Third, the CRC patients who participated in our study lacked information about treatment therapy, including surgery and neoadjuvant chemotherapy, which prevented us from exploring the influences of selected biomarkers on the quality of treatment outcomes. In addition, the follow-up information must be improved to determine whether these selected metabolites predict the prognosis of colorectal cancer patients.

In conclusion, we analysed the differential serum metabolites between CRC patients and healthy controls by metabonomics analysis on an LC-MS platform and identified four potential metabolic biomarkers that could discriminate CRC patients: increased hexadecanedioic acid, 4-dodecylbenzenesulfonic acid, and formylanthranilic acid, in parallel with decreased 2-pyrocatechui acid. More importantly, we found two crucial differential metabolic pathways: the vitamin B metabolic pathway and the alanine, aspartate, and glutamate metabolism pathway, both of which were activated in CRC patients. Thus, metabolomics analysis is a promising approach to investigate tumour biomarkers.

## Figures and Tables

**Table 1:** Demographic characteristics of the included subjects.

	CRC group *N* = 98	Control group *N* = 50	*p* value
Male/female	64/34	32/18	
Age (year, mean ± SD)	61.61 ± 9.86	61.52 ± 6.81	0.955
BMI (kg/m^2^, mean ± SD)	24.56 ± 3.31	25.13 ± 3.01	0.309
CEA (ng/mL, mean ± SD)	8.91 ± 20.07	3.34 ± 1.85	0.085
Cancer location			
Colon (number)	41	—	
Rectum (number)	57	—	

**Table 2:** The identified differential metabolites between the CRC group and the healthy control.

Name	KEGG Compound ID	RT	*M*/*Z*	VIP	*p* value	Fold change	Log_fold change
3-Hydroxybutyric acid	C01089	243.25	103.04	1.56	0.001	2.01	1.007
Hexadecanedioic acid	C19615	206.06	285.21	1.53	0.018	1.73	0.7923
Succinic acid semialdehyde	C00232	82.39	101.02	1.46	0.014	1.25	0.316
4-Dodecylbenzenesulfonic acid	N/A	28.15	325.18	1.62	1.06301*E* − 06	1.64	0.711
Prostaglandin B2	C05954	178.14	333.21	1.08	0.034	1.26	0.335
2-Pyrocatechuic acid	C00196	61.20	153.02	1.86	0.0002	0.49	-1.042
Xanthoxylin	C10726	148.02	195.07	1.38	0.049	0.49	-1.014
12-Hydroxydodecanoic acid	C08317	71.41	215.17	1.03	0.002	1.33	0.417
Formylanthranilic acid	C05653	71.61	164.03	1.47	1.22427*E* − 06	2.084	1.059

**Table 3:** Results of the topological analysis and enrichment analysis of differential metabolites.

Pathway	Total	Hits	Raw *p*	-ln(*p*)	Impact
Alanine, aspartate, and glutamate metabolism	24	1	0.149	1.907	0.057
Vitamin B6 metabolism	32	1	0.193	1.644	0
Butanoate metabolism	40	1	0.236	1.445	0.033
Arachidonic acid metabolism	62	1	0.342	1.072	0
Tyrosine metabolism	76	1	0.402	0.910	0.005
Tryptophan metabolism	79	1	0.415	0.880	0.008

## Data Availability

The raw data required to reproduce these findings cannot be shared at this time as the data also forms part of an ongoing study.
